# Supplementary material for: WordCommentsAnalyzer: A windows software tool for qualitative research
Source: F1000Res. 2018 Sep 4;7:536. Originally published 2018 May 3. [Version 2] doi: 10.12688/f1000research.14819.2 (PMC6137411; doi:10.12688/f1000research.14819.2)
Supplement: Supplementary file 3 [file f1000research-7-17614-s0002.tgz › b2a79496-4ada-47c4-9682-a99ce867836d.zip!/Eight Iranian Health Journals/Journal of Health System Research _14.docx]

© Journal of Health System Research . This work is licensed under CC BY-NC 4.0 (https://creativecommons.org/licenses/by-nc/4.0/).

Volume & Issue: دوره 10، شماره 4، 1393

Title: ارتباط شاخص گليسميك و بار گليسميك رژيم غذايي با بيماريهاي قلبي عروقي: مروري بر شواهد اپيدميولوژيك

http://hsr.mui.ac.ir/index.php/jhsr/article/view/1437

مقدمه: سبک زندگی افراد جامعه خصوصا عوامل غذایی نقش مهمی در افزایش روزافزون بیماری‌های قلبی و عروقی دارد. با توجه به دریافت‌های غذایی کربوهیدرات به‌ویژه در کشورهای در حال توسعه، توجه به نوع کربوهیدرات مصرفی در بروز بیماری‌های قلبی و عروقی کمتر مورد توجه قرار گرفته است. مطالعه حاضر با هدف مروری بر مطالعات پیشین در زمینه ارتباط بین نمایه گليسميک و بار گليسميک رژیم غذایی با بیماری‌های قلبی و عروقی انجام گرفت. روش‌ها: با استفاده از موتور جستجوي  Pubmedو کلیدواژه‌هایی مانند glycemic index ،  glycemic load،cardiovascular disease وcoronary heart disease  مقالات با طراحی‌هاي مقطعی، کارآزمایی بالینی، کوهورت آینده‌نگر انتخاب شدند. یافته‌ها: نتایج مطالعات ناهمسو است اما نشان می‌دهد دریافت رژیم غذایی با نمایه و بار گليسميك پایین دارای اثرات مفیدی بر متابولیسم گلوکز و غلظت چربی‌های سرم چربی‌ها در بدن می‌باشد و به عنوان یک عامل محافظتی در بیماری‌های قلبی و عروقی مطرح می‌شود. نتیجه‌گیری: شواهد موجود رابطه‌ای قطعی بین نمایه گليسميک و بار گليسميک با بيماري‌هاي قلبي عروقي را گزارش نکرده‌اند. انجام مطالعات بیشتر و دقیق‌تر ضروری به نظر می‌رسد.

Title: ارزيابي تغذيه اي بيماران بستري در بخش مراقبت هاي ويژه بيمارستان الزهرا در اصفهان در سال 1390

http://hsr.mui.ac.ir/index.php/jhsr/article/view/985

مقدمه: سوء‌تغذيه از عوامل مهمي است که مدت بستري در بيمارستان را افزايش مي‌دهد، از طرفي تغذيه مناسب، نتايج باليني بيماران بدحال را بهبود مي‌بخشد. با توجه به انجام ارزيابي‌هاي اندک در اين زمينه، در این مقاله بر آن هستیم تا به ارزيابي شاخص‌هاي آنتروپومتريک و بيوشيميايي‌ بيماران بستري در بخش مراقبت‌هاي ويژه بپردازیم. روش‌ها: این مطالعه بر روی 100 بیمار بستری در بخش مراقبت ویژه بیمارستان الزهرا در اصفهان انجام شد. اندازه‌گيري‌ها شامل قد، وزن، انرژي دريافتي، انرژي مورد نياز فرد و هم‌چنين اندازه‌گيري محيط ميانه‌ي دور بازو و دماي بدن بود. شاخص‌هاي بيوشيميايي نيز دو مرتبه اندازه‌گيري شده‌اند. شاخص‌هاي کيفي اندازه‌گيري شده شامل نوع بيماري، عفونت، زخم بستر، تهويه و نوع تغذيه‌ي بيمار بود. تحليل داده‌ها با استفاده از نرم‌افزارSPSS16  صورت گرفته است. یافته‌ها: انرژي دريافتي در 61% در اين بيماران کمتر از انرژي مورد نياز آن‌ها مي‌باشد و تنها 39% از آن‌ها انرژي کافي را دريافت کرده‌اند. سطح سرمي پتاسيم، فسفر و ميزان آلبومين سرم در محدوده‌ي طبيعي و ميانگين کراتينين بالاتر از محدوده‌ي طبيعي قرار داشت. ميزان دور بازو در 69% بيماران نسبت به اندازه‌گيري روز اولشان کاهش يافت. بيشترين نوع تغذيه در بيماران مربوط به تغذيه با گاواژ بود (54%). اصلي‌ترين علت بستري شدن، انواع جراحي به‌خصوص جراحي پس از سوانح مانند حوادث رانندگي بوده است. نتیجه‌گیری: بيماران بستري در بخش ICU  در معرض سوء تغذيه بيمارستاني مي‌باشند و نيازمند مراقبت‌هاي ويژه تغذيه‌اي هستند.

Title: شاخص فشار نسبی : ارزیابی ریسک مشاغل صنایع نساجی از دیدگاه ماکروارگونومی

http://hsr.mui.ac.ir/index.php/jhsr/article/view/1051

مقدمه: ارزیابی ریسک ماکروارگونومی یکی از روش‌های ارزیابی ریسک ارگونومی جهت شناسایی انواع خطراتی می‌باشد که افراد در محیط‌های کاری با آن‌ها مواجهه هستند. شاخص فشار نسبی یکی از روش‌های ارزیابی ریسک ماکروارگونومی است که قادر به شناسایی ریسک فاکتورهای مرتیط با بروز مخاطرات شغلی در محیط کار می‌باشد. هدف از انجام این مطالعه ارزیابی ریسک ارگونومیکی مشاغل موجود در صنایع نساجی با شاخص فشار نسبی بود. روش‌ها: این مطالعه به روش سرشماری و در بین156 نفر از پرسنل شاغل درصنایع نساجی در سه شیفت کاری صبح، عصر و شب انجام گرفت. جهت محاسبه شاخص فشار نسبی،معادل‌های گوناگونی برای اجزای کار تعریف شده که توسط آن‌ها این شاخص محاسبه می‌گردد. ارزش نهايي براي شاخص فشار نسبی،يك معيار بین صفر تا 10 می‌‌باشد که عدد صفر بر خطرناک/ ناايمن/ بدون بهره‌ور بودن و عدد 10 بر بي خطر/ ايمن/ بهره ور بودن دلالت دارد. یافته‌ها: آزمون آماری ANOVA نشان داد که در بین 6 حیطه شاخص فشار نسبی، حیطه‌ی کار دستی و حواس در بین شیفت‌های کاری مختلف در صنایع نساجی دارای ارتباط معنی‌داری با خطر بروز مخاطرات شغلی می‌باشند (05/0P<). هم‌چنین حیطه‌های پوسچر و حمل دستی بار به ترتیب با میانگین (11/0)19/6 و (41/0)14/9 کمترین و بالاترین نمره را در بین 6 حیطه شاخص فشار نسبی به خود اختصاص دادند. نتیجه‌گیری: براساس یافته‌های حاصل از پژوهش، وضعیت بدنی افراد در هنگام کار و کار دستی به‌عنوان تأثیرگذارترین عوامل در بروز مخاطرات شغلی در بین افراد شاغل در صنایع نساجی شناخته شدند. لذا حفظ پوسچر مناسب در هنگام فعالیت وکاهش میزان فعالیت‌هایی دستی توسط افراد در طی هر شیفت کاری می تواند تأثیر به‌سزایی در کاهش مخاطرات شغلی داشته باشد.

Title: جنسیت و مهارت‌های چهارگانه هوش هیجانی

http://hsr.mui.ac.ir/index.php/jhsr/article/view/1098

مقدمه: هوش هیجانی به عنوان توانایی درک احساسات خود و دیگران، ارتباط با مردم، انطباق و کنار آمدن با فشارها وخواسته‌های محیطی تعریف می‌شود. مهارت‌هایی چون خودآگاهی هیجانی،خودمدیریتی،آگاهی اجتماعی،مدیریت روابط به عنوان چهار مهارت متشکل هوش هیجانی شناخته شده‌اند. روش‌ها: این مطالعه توصیفی همبستگی به صورت مقطعی در سال تحصیلی 92-91 روی 248 نفر(137پسر و111 دختر) از مجموع 740 نفر از دانشجویان دختر وپسر موسسه آموزش عالی باختر مرکز ایلام که با استفاده از روش نمونه‌گیری طبقه‌ای انتخاب شدند اجرا گردید. جهت بررسی نقش جنسیت در مهارت‌های چهارگانه هوشی هیجانی از پرسش‌نامه هوش هیجانی (Bradbry Travis - Graves) 28 سؤالی استفاده گردید. تجزیه و تحلیل داده‌ها با استفاده از نرم‌افزارspss  نسخه 20 و آزمون‌های آماری ضریب همبستگی پیرسون،تحلیل multivariate صورت گرفت. یافته‌ها: نتایج پژوهش نشان داد که بین میزان هوش هیجانی و مهارت‌های چهارگانه آن (خودآگاهی هیجانی، خودمدیریتی، آگاهی اجتماعی، مدیریت روابط) دختران و پسران تفاوت معنی‌داری وجود دارد و میانگین نمرات هوش هیجانی و مهارت‌های چهارگانه آن در دختران بیشتر از پسران است. نتیجه‌گیری: جنسیت با هوش هیجانی در ارتباط بوده و از سویی میزان مهارت‌های هوش هیجانی در دختران و پسران می‌تواند با هم تفاوت داشته باشد.

Title: مقایسه سیستم‌های آبرسانی توزیع ثقلی و پمپاژ مستقیم از نظر فشار، نشت و کیفیت آب با استفاده از مدل water Gems v3/0 ( مطالعه موردی شبکه آبرسانی شهر بروجن )

http://hsr.mui.ac.ir/index.php/jhsr/article/view/1348

مقدمه: فشار نقاط مصرف در شبکه‌های آبرسانی یکی از مهم‌ترین پارامترهای هیدرولیکی است که می‌تواند در مدیریت بهینه شبکه‌های توزیع آب مورد استفاده قرار گیرد. از آن‌جایی که فشار، اثرات متفاوتی بر پارامترهای مختلف مدیریت شبکه، هم‌چون عملکرد هیدرولیکی، قابلیت اطمینان، پایداری شبکه و نشت دارد لذا کنترل آن از اهمیت بسیاری در سطوح مختلف مدیریتی برخوردار است. در این تحقیق سعی شده با اجرای برنامه برای دوشبکه توزیع آب شهری وضعیت فشار، نشت و کیفیت آب در نقاط مختلف شبکه مورد مقایسه و تجزیه و تحلیل قرار گیرد. روش‌ها: به‌منظور مدل‌سازی شبکه از نرم‌افزار  water Gems v3/0 استفاده شد. با استفاده از این نرم‌افزار، شبکه به دو صورت ثقلی و پمپاژ مستقیم، مدل و طراحی گردید. یافته‌ها: در زمان مینیمم مصرف، فشار در 45 درصد از گره‌های شبکه توزیع ثقلی بالای 70 متر می‌باشد، در حالی که در پمپاژ مستقیم به جز دو گره، فشار از 60 متر تجاوز نکرد. در زمان ماکزیمم مصرف فشار در 15 درصد از گره‌های شبکه توزیع ثقلی و 35% از شبکه پمپاژ مستقیم کمتر از 30 متر می‌باشد. میزان نشت آب در شبکه توزیع ثقلی در زمان مینیمم مصرف 43% بیشتر از حالت مشابه در پمپاژ مستقیم می‌باشد. نتایج نشان داد که میزان کلر باقی‌مانده در 62% از لوله‌های شبکه پمپاژ مستقیم در حد استاندارد و در 100% از لوله‌های موجود در شبکه توزیع ثقلی کمتر از حد استاندارد بود. نتیجه‌گیری: در اين مطالعه با مقايسه پارامترهای فشار، میزان نشت، سن آب و میزان کلر باقیمانده، بين دو سیستم توزیع ثقلی و استفاده از ايستگاه‌هاي پمپاژ مستقیم آب، نشان داده شد که کلیه این پارامترها در شبکه پمپاژ مستقیم در شرایط مطلوب‌تر و نزدیک‌تر به مقادیر استاندارد آن می‌باشند.

Title: ارزیابی کارایی جاذب هلمهولتز طراحی شده در کاهش صدای فرکانس پایین ناشی از کانال های هوا و سیستم‌های تهویه

http://hsr.mui.ac.ir/index.php/jhsr/article/view/1535

مقدمه: با توجه به عوارض غیر قابل جبران سر و صدا در جامعه و محیط‌های صنعتی، کاهش آلودگی صوتی در منبع تولید صوت بهترین و مؤثرترین راه کنترلی است. فیلتر آکوستیکی ) سایلنسر( ابزاری است که از انتقال امواج صوتی جلوگیری می‌کند. نوعی از این سایلنسر، جاذب هلم هولتز (تقویت‌کننده با شاخه فرعی) می‌باشد که برای فرکانس‌های پایین و زیر 200 هرتز مانند صدای فن و کانال‌ها مناسب می‌باشد. هدف این مقاله بررسی کارایی جاذب طراحی شده در کاهش صدای‌های پایین می‌باشد. روش‌ها: این مطالعه از نوع تجربی است. جاذب هلمهولتز طراحی شده روی کانالی مشابه کانال تهویه نصب شد. با استفاده از نرم افزار پردازش و تولید سیگنال (MATLAB) ، سیگنال‌هایی در فرکانس‌های بسیار پایین 250 تا 500 هرتز تولید و از طریق بلندگو در طول کانالی پخش شد. سپس با مقایسه تراز فشار صوت قبل و بعد از جاذب هلمهولتز میزان صوت کاهش یافته را در فرکانس‌های مختلف بررسی و پردازش سیگنال‌های داخل کانال و تهیه الگوریتم صدا در کانال توسط نرم‌افزار MATLAB انجام شد. یافته‌ها: بیشترین کاهش صدا در فرکانس 125 هرتز در بین جاذب‌های هلمهولتز، با قطر محفظه 63 میلی متر و قطر دهانه جاذب 1 سانتی متر بود. میزان کاهش صدا در فرکانس 125 هرتز بیشترین و 500 هرتز کمترین بود. با توجه به اندازه‌گیری‌های انجام شده در فرکانس 125 هرتز با افزایش صدا توسط جاذب‌ها و هم‌چنین با افزایش قطر دهانه جاذب، میزان جذب صدا کاهش می‌یابد. نتیجه‌گیری: این پژوهش نشان داد که جاذب‌های هلمهولتز طراحی و نصب شده در کانال در فرکانس مشخصی از صدای با فرکانس‌های پایین بیشترین کاهش صوتی را خواهد داشت که این کاهش در جاذب‌های با سایز کوچکتر مشهودتر بود و هم‌چنین با افزایش قطر محفظه جاذب‌های هلمهولتز و افزایش سطح مقطع گردنه در جاذب‌ها میزان جذب صدا کاهش پیدا کرد. با توجه به این‌که استفاده بیشتر از وسایل جدید در محیط‌های عمومی نظیر سیستم‌های تهویه، کمپرسورها، رایانه، پرینتر و غیره مشکلات دیگری را در ارتباط با صدای با تراز نه چندان بلند اما آزاردهنده در فرکانس‌های کم ایجاد نموده است، در نتیجه با تعیین محدوده فرکانسی مؤثر جاذب هلمهولتز، می‌توان از آن به عنوان وسیله‌ای مؤثر و کارامد جهت کاهش یا حذف صدا استفاده نمود.

Title: رضایت‏مندی اعضای نهادهای محلی استان‏های شمالی ایران از مراکز مجری برنامه پزشک خانواده و بیمه روستایی

http://hsr.mui.ac.ir/index.php/jhsr/article/view/1575

چكيده   مقدمه: جلب همکارهای برون‏بخشی یکی از استراتژی‏های بهداشت برای همه تا سال 2000 بوده که در قرن بیست‏و‏یکم هم مورد تأکید قرار گرفت. اگر اعضای نهادهای محلی از واحدهای ارایه‏دهنده خدمات سلامت راضی‏تر باشند، همکاری و مشارکت بیشتری در اعتلای برنامه‏های سلامت می‏نمایند. این مطالعه به منظور تعیین سطح رضایت اعضای نهادهای محلی از مراکز مجری برنامه پزشک خانواده و بیمه روستایی در استان‏های شمالی ایران در سال 1391 انجام گرفت. روش‌ها: مطالعه از نوع توصیفی-تحلیلی بوده که به صورت مقطعي در شش ماهه دوم 1391 انجام گرفت. 25% مراکز مجری طرح پزشک خانواده و بیمه روستایی در سه استان گلستان، مازندران و گیلان، مجموعا 139 مرکز از 552 مرکز مجری بصورت تصادفی منظم به عنوان خوشه‏های پژوهش انتخاب و در هر مرکز منتخب اعضای شورای حل اختلاف، شورای اسلامی، شورای بهداشتی، دهیار، مدیر و معلم بومی در دست‏رس مورد بررسی قرار گرفتند. ابزار جمع‏آوری داده‏ها پرسش‌نامه محقق ساخته بوده که روایی و پایایی آن مورد تائید قرار گرفت. داده‏ها در SPSS17 در سطح معنی‏داری P<0.05 تحلیل شد. یافته‌ها: از بین 601 عضو نهادهای محلی مورد مطالعه، 521 نفر (7/86 درصد) مرد بودند. میانگین و انحراف معیار سنی کل افراد 7/9±45 سال بود. میانگین سطح رضایت اعضای نهادهای محلی از خانه بهداشت از 5 نمره، 6/0±3/4 و از مرکز بهداشتی درمانی 7/0±7/3 بوده است. پایین‏ترین سطح رضایت مربوط به کامل نبودن امکانات و خدمات قابل ارائه و بالا بودن هزینه خدمات دریافت شده بوده است. سطح رضایت اعضای نهادهای محلی از خانه‏های بهداشت در استان‏های شمالی ایران تفاوت معنی‏دار داشت (005/0=P). بین نوع نهاد محلی و رضایت کلی از خانه بهداشت و مرکز بهداشتی درمانی رابطه معنی‏دار وجود نداشت (149/0=P)، (893/0=P). نتیجه‌گیری: این مطالعه نشان داد اعضای نهادهای محلی از واحدهای سطح اول نظام شبکه به جزء در 2 مورد (کامل بودن امکانات و هزینه خدمات) راضی بودند. پیشنهاد می‏گردد شبکه بهداشت و درمان شهرستان‏ها با جلب مشارکت اعضای نهادهای محلی از ظرفیت‏های موجود جهت اعتلای سلامت بهره‏برداری نمایند.

Title: اندازه‌گیری فاکتورهای مؤثر بر نیروی چنگش بر اساس استاندارد پیشنهادی

http://hsr.mui.ac.ir/index.php/jhsr/article/view/1612

مقدمه: اندازه‌گیری نیروی چنگش یکی از پارامترهایی است که در پزشکی و علم ارگونومی کاربرد دارد. این مطالعه دارای 2 هدف اصلی بود. اول تعیین رابطه بین عوامل آنتروپومتریک بر قدرت چنگش دست، دوم اولویت‌بندی این متغیرها در پیش‌بینی قدرت چنگش دست. روش‌ها: این مطالعه از نوع کاربردی بوده و بر روی 204 نفر انجام شد و 14 بعد دست با استفاده از کولیس دیجیتالی (ساخت شرکت Mitutoyo، ژاپن) با رزولوشن 01/0 میلی‌متر و دقت 01/0 میلی‌متر اندازه‌گیری شد. قدرت چنگش در کلیه افراد شرکت‌کننده در این پژوهش به وسیله دستگاه داینامومتر مدل jamar مطابق توصیه انجمن درمانگران دست آمریکا (ASHT یا American Society of Hand Therapists) اندازه‌گیری شد. یافته‌ها: نتایج نشان دادند که بین قدرت چنگش و ابعاد دست رابطه مستقیم وجود دارد. ضریب همبستگی پیرسون نشان می‌دهد رابطه مستقیمی بین ابعاد دست، طول قد، شاخص توده بدن و قدرت چنگش وجود دارد و دست غالب به‌طور معنی‌داری قوی‌تر از دست دیگر بود.فاکتور قد با ضریب همبستگی 63/0 و بعد از آن بیشترین پهنای دست (عمودی) با ضریب همبستگی 33/0 در قدرت چنگش نقش به‌سزایی داشته است. نتیجه‌گیری: از آن‌جا که وسیله‌ی اندازه‌گیری قدرت چنگش در همه صنایع وجود ندارد لذا به راحتی می‌توان از پارامترهای قد و پهنای دست برای پیش‌بینی قدرت چنگش دست افراد استفاده نمود.

Title: آگاهی و باورهای زنان در خصوص ماموگرافی بر اساس سازه های مدل اعتقاد بهداشتی

http://hsr.mui.ac.ir/index.php/jhsr/article/view/1616

مقدمه: سرطان پستان شايع‌ترين سرطان مهاجم و دومين علت مرگ ناشي از سرطان در اكثر كشورهاي دنيا محسوب مي‌شود. انجام روش‌هاي غربالگري در تشخيص اوليه سرطان پستان بسيار مهم مي‌باشد. اين مطالعه به بررسي آگاهی و باورهاي بهداشتي زنان بالاي40 سال در خصوص انجام ماموگرافي پرداخته است. روش‌ها: این پژوهش یک مطالعه توصیفی مقطعی می‌باشد که بر روی 384 نفر از زنان مراجعه‌کننده به مراکز بهداشتی درمانی در سال 90-1389 انجام گرفت. نمونه‌ها از طریق نمونه‌گیری غیر تصادفی در دسترس انتخاب شدند و اطلاعات به وسیله پرسش‌نامه، به صورت خودگزارش‌دهی جمع‌آوری گردید. داده‌ها با استفاده از نرم‌افزار آماری SPSS و استفاده از آمار توصیفی و آزمون کای دو مورد تجزیه وتحلیل قرار گرفت. یافته‌ها: نتایج نشان داد که اكثريت زنان مورد مطالعه ( 9/86% ) آزمون غربالگري ماموگرافي را انجام نداده بودند. آگاهی 7/48% افراد در زمینه سرطان پستان در حد متوسط بود و 2/37% آن‌ها معتقد بودند دلیل انجام ندادن ماموگرافی، عدم توصیه پزشک به آنان می‌باشد. هم‌چنین بین میزان تحصیلات و آگاهی واحدهای پژوهش ارتباط معنی‌داری دیده شد (001/0=P). بین میزان تحصیلات و شدت درک شده (004/0>P) ، منافع درک شده (001/0>P) و موانع درک شده (001/0>P) ارتباط معنی‌دار وجود داشت. در صورتی که بین میزان تحصیلات وحساسیت درک شده ارتباط معنی‌داری (03/0=P) دیده نشد. نتیجه‌گیری: نتایج این مطالعه لزوم توجه بیشتر برنامه‌ریزان بهداشت و درمان کشور را به آموزش و ترویج روش‌های غربالگری و برطرف نمودن موانع آشکار می‌سازد. هم‌چنین پیشنهاد می‌شودکه ماموگرافی به طور روتین و رایگان به عنوان یک آزمون غربالگری در زنان بالای 40 سال مراجعه‌کننده به مراکز بهداشتی به اجرا گذاشته شود.

Title: ارتباط میان گونه هواها‌ی شهر اصفهان و مرگ و میر ناشی از بیماری‌های دستگاه تنفسی

http://hsr.mui.ac.ir/index.php/jhsr/article/view/1692

مقدمه: سلامتی در جامعه به عنوان سرمایه‌گذاری شناخته می‌شود و آب‌ و ‌هوا، از تأثیرگذارترین عوامل بر سلامتی است. هدف از این مطالعه تعیین گونه‌ هواها و رابطه‌ی هر یک از آن‌ها با مرگ‌ومیر ناشی از بیماری‌های سیستم تنفسی در اصفهان می‌باشد. روش‌ها: در این پژوهش، با استفاده از 44 متغیر اقلیمی، در یک دوره پنج‌ساله و از طریق نرم‌افزار MATLAB، گونه هواهای غالب اصفهان مشخص گرديد. پراکندگی زماني گونه‌ هواها و هم‌چنین مرگ‌ومیر در اصفهان تعیین شد. نهايتا، پهنه‌بندی بیماری‌هاي سیستم تنفسی در مناطق شهرداری اصفهان با استفاده از نرم‌افزارGIS  Arc مشخص گردید. یافته‌ها: اصفهان هفت‌گونه هوا داردکه احتمال مرگ‌ومیر ناشی از بیماری مزمن ریه و برنش و آمبولی ریه به ترتیب با 5/27‌% و 2/26‌% در هنگام رخداد گونه گرم و خشک و احتمال مرگ بر اثر پنومونی و آسم به ترتیب با 09/23 و 7/21 درصد در هنگام رخداد گونه سرد، خشک، کم‌بارش، آرام و احتمال مرگ بر اثر بسته شدن راه‌های تنفسی با 6/66‌% در هنگام رخداد گونه‌ی سرد بادی است. اوج حاکمیت این گونه‌ها در ماه‌های تیر‌، شهریور، دی و بهمن می‌باشد و از نظر پراکندگی مکانی مرگ‌ومیر ناشی از این دو بیماری در مناطق شهرداری اصفهان متفاوت است. نتیجه‌گیری: افرادی که بیماری‌های تنفسی دارند بهتر است در اوج حاکمیت گونه‌هواهای سرد و خشک ، سرد بادی و گرم و خشک یعنی اواسط ماه‌های شهریور ‌و تیر هم‌چنین دی و بهمن مراقبت بیشتری شوند و مسؤولین باید در برنامه‌ریزی‌هاي آينده به بعضی از مناطق شهرداری اصفهان توجه بیشتری کنند.

Title: بررسی تأثیر مداخله آموزشی بر اضطراب پرستاران شاغل در بیمارستان‌های وابسته به دانشگاه علوم پزشکی شهر اراک: کاربرد مدل پرسید

http://hsr.mui.ac.ir/index.php/jhsr/article/view/1713

مقدمه: افراد مشغول به كار در مشاغلي مانند پرستاري داراي استرس فراوان و در معرض ابتلا به اختلالات اضطرابي(Anxiety disorders) مي‌باشند. هدف این مطالعه تعیین تأثیر برنامه آموزشی آرام‌سازی کاربردی براساس PRECEDE model بر کاهش اضطراب پرستاران در سال 1392-1391بود. روش‌ها: در این مطالعه مداخله‌اي با گروه شاهد، 40 کارشناس پرستاری از بیمارستان حضرت ولی عصر (عج) در گروه مداخله و 40 کارشناس پرستاری از بیمارستان حضرت امیرالمومنین (ع) شهر اراک در گروه شاهد قرار داشتند، گروه مداخله و شاهد به صورت تخصیص تصادفی انتخاب شدند. جهت سنجش اضطراب از پرسش‌نامه (State-Trait Anxiety Inventory) Spielberger و جهت مداخله از پرسش‌نامه طراحی شده براساس PRECEDE model استفاده گردید. مداخله به مدت 7 جلسه و هر جلسه 60 تا 90 دقیقه انجام گردید. تکمیل پرسش‌نامه قبل، بلافاصله و شش ماه بعد از مداخله صورت گرفت. داده‌ها در نرم‌افزار آماری SPSS نسخه 20 مورد تجزیه و تحلیل قرار گرفت. مقدار( 05/0p< )معنی‌دار در نظر گرفته شد. یافته‌ها: میانگین نمره اضطراب آشکار و پنهان قبل از مداخله بین دو گروه اختلاف معنی‌دار نداشت (3/0=P ). اما بلافاصله بعد از مداخله و نیز 6 ماه بعد از مداخله میانگین نمره اضطراب آشکار و پنهان در گروه مداخله به طور معنی‌دار کمتر از گروه شاهد شد (001/0P<). بلافاصله و نیز 6 ماه بعد از مداخله میزان عوامل مستعدکننده، قادرکننده، تقویت‌کننده و رفتارهای مقابله با اضطراب در گروه مداخله نسبت به شاهد افزایش معنی‌دار داشت (001/0P<). نتیجه‌گیری: یافته‌ها تأثیر برنامه آموزشی بر اساس الگوی پرسید را جهت کاهش اضطراب پرستاران تأیید می‌نماید. به نظر می‌رسد استفاده از مداخله‌های آموزشی مبتنی بر الگوی پرسید (به خصوص آموزش‌های بهبود سلامت جسمی و روحی) بتواند در جمعیت‌های خاص و در کل جامعه باعث ارتقای سلامت و افزایش کارایی جامعه مورد نظر شود.

Title: بررسی کارایی خاک مونتموریونیت در جذب سدیم دودسیل سولفات از محلول‌های آبی

http://hsr.mui.ac.ir/index.php/jhsr/article/view/1753

چكيده   مقدمه: امروزه مقادیر زیادی سورفاکتانت‌های حاوی SDS مصارف خانگی داشته و در انتهای جریان فاضلاب شهرها و ورودی تصفیه خانه‌ها دیده می‌شوند. هدف از مطالعه حاضر بررسی کارایی جذب سورفاکتانت سدیم دودسیل سولفات بر خاک مونتموریونیت به عنوان یک جاذب ارزان قیمت و در دسترس در ایران بوده است. روش‌ها: اين تحقيق یک مطالعه تجربی است اثر غلظت اولیه سدیم دودسیل سولفات (SDS) ، جرم جاذب و زمان واکنش بر حذف سدیم دودسیل سولفات توسط جاذب خاک مونتموریونیت مورد بررسی قرار گرفت. مطالعه روي نمونه‌های سنتزی حاوي غلظت‌هاي 30،10 و50 ميلي‌گرم در ليتر دودسیل سولفات انجام گرفت. غلظت مقادير سدیم دودسیل سولفات به روش اسپكتروفتومتري در طول موج 625 نانومتر تعیین شد. مقادیر 1/0، 25/0و 5/0 گرم در لیتر جرم جاذب در pH ( 4 ، 7 و10) در شرایط اختلاط کامل هوا با زمان های تماس (5/2 ، 5، 10، 20 و 30) دقیقه مورد آزمایش قرار گرفت. مقادیر SDS و COD در مراحل مختلف اندازه گیری شد. یافته‌ها: : با افزايش جرم جاذب و زمان واکنش مقدار حذف آلاینده افزایش و با افزایش غلظت اولیه دودسیل سولفات مقدار حذف کاهش یافت. داده‌های این تحقیق نشان داد که بیشترین میزان جذب دودسیل سولفات توسط خاک مونتموریونیت در مدت زمان 30 دقیقه انجام و آزمایش به حالت تعادل می‌رسد. بیشترین میزان راندمان حذف در شرایط بهینه (غلظت اولیه mg/l 10، جرم جاذب 5/0 گرم در لیتر و 7pH =  اتفاق افتاد. راندمان حذف SDS و CODبه ترتیب برابر با 5/77 و76 درصد بوده است. نتیجه‌گیری: بررسی راندمان جذب SDS بر ذرات خاک مونتموریونیت بیانگر تغییرات جزئی در pH های مختلف بوده به نحوی که با افزایش pH از 4 به 10 راندمان به مقدار کمی افزایش یافته است. فرایند جذب هم‌چنین تحت تأثیر جرم جاذب و غلظت اولیه SDS نیز قرار داشته به نحوی که افزایش جرم جاذب وکاهش غلظت اولیه آلاینده موجب افزایش کارایی جذب شده است.

Title: بررسی ارتباط بار کاری ذهنی با اختلالات اسکلتی عضلانی در بین پرستاران بیمارستان الزهرا با استفاده از شاخص بار كاري NASAو CMDQ

http://hsr.mui.ac.ir/index.php/jhsr/article/view/1834

مقدمه: بارکاری ذهنی یک مفهوم کلی در ادبیات ارگونومی و فاکتورهای انسانی و یک ساختار پیچیده و چند بعدی است که به وسیله نیازهای کاری خارجی، محیط، فاکتورهای روانی و سازمانی، توانایی‌های ذهنی و سازمانی تحت‌تأثیر قرار می‌گیرد. هدف از این مطالعه ارزیابی بارکاری ذهنی و تأثیر آن روی ناراحتی‌های اسکلتی عضلانی در بین پرستاران شاغل در بخش‌های مختلف بیمارستان مي‌باشد. روش‌ها: این يك مطالعه توصیفی - تحلیلی از نوع مقطعی بود كه روي 247 نفر از پرستاران مرکز آموزشی درمانی الزهرا اصفهان كه به طور تصادفي سيستماتيك انتخاب شدند، در تابستان 1392 انجام گرفت. جهت جمع‌آوري داده‌ها از نسخه فارسي پرسش‌نامه تخصصي NASA-Tlx (National Aeronautics and Space Administration Task Load Index) ( براي اندازه‌گيري بار ذهني) و از پرسش‌نامه ناراحتی‌های اسکلتی - عضلانی کرنل CMDQ)) استفاده شد. داده‌های گردآوری شده با استفاده از نرم‌افزار آماری SPSS20 و به وسيله آزمون همبستگي پيرسون، اسپيرمن و رگرسيون خطي چندگانه مورد تجزیه و تحلیل قرار می‌گیرد. یافته‌ها: ضريب همبستگي پيرسون نشان داد كه بين ميزان ناراحتي‌هاي اسكلتي عضلاني پرستاران به ترتيب با ابعاد باركاري نااميدي، باركاري كل، نياز زماني، تلاش و نياز فيزيكي رابطه معني‌داري وجود داشت( 211/0، 216/0، 277/0، 277/0، 304/0 (r=  ولي بين ابعاد باركاري عملكرد و نياز ذهني با شيوع ناراحتي هاي اسكلتي- عضلاني در ميان پرستاران رابطه مستقيم وجود نداشت (05/0p< 304/0r= ) نتیجه‌گیری: ميزان نااميدي پرستاران ارتباط مستقيمي با ناراحتي‌هاي اسكلتي عضلاني داشت. اين نشان مي‌دهد كه استرس يك جز جدايي‌ناپذير در محيط‌هاي كاري بيمارستان‌ها مي‌باشد. بنابراين بايستي يكي از اولويت‌هاي اصلي مديران بيمارستان‌ها كاهش استرس در محيط كار پرستاران باشد

Title: شناسایی خوشه‌های پرخطر موارد بروز سرطان معده در ایران با استفاده از آماره کاوشی فضا-زمان طی سال‌های 1388-1384

http://hsr.mui.ac.ir/index.php/jhsr/article/view/2072

مقدمه: سرطان معده دومین سرطان شایع و کشنده‌ترین سرطان در ایران به شمار می‌آید. در اين پژوهش به پهنه‌بندی و توزیع جغرافیایی سرطان معده و شناسایی خوشه‌های پرخطر این سرطان با استفاده از آماره کاوشی فضا- زمان پرداخته شده است. روش‌ها: اين مطالعه از نوع كاربردي بود. داده‌های مورد نیاز آن از گزارش‌های ثبت سرطان اداره سرطان مرکز مدیریت بیماری‌های وزارت بهداشت، درمان و آموزش پزشکی استخراج شد. این داده‌ها به تفکیک استان‌ها برای یک دوره 5 ساله از سال 1384 تا 1388 ثبت شده بودند. برای تجزیه و تحلیل اطلاعات و شناسایی خوشه‌ها از نرم‌افزار SaTScan و برای پهنه‌بندی توزیع سرطان معده و نمایش خوشه‌ها در کشور از نرم‌افزارArcGIS10  استفاده شد. یافته‌ها: پس از تجزیه و تحلیل سه خوشه پرخطر شناسایی گردید. محتمل‌ترين خوشه، استان‌های اردبيل، گيلان، زنجان، آذربايجان‌شرقي، قزوين، آذربايجان‌غربي، كردستان، همدان، تهران و مازندران را در دوره زماني 1387 تا 1388 شامل گردید که از لحاظ آماری در سطح 05/0 معنی‌دار بود. علاوه بر محتمل‌ترين خوشه، دو خوشه‌ی ثانويه نيز به دست آمد. خوشه‌ی ثانويه اول، شامل استان‌هاي خراسان جنوبي، كرمان و خراسان رضوي و از لحاظ آماری معنی‌دار به دست آمد. خوشه ثانویه دوم استان کهگیلویه و بویراحمد را در بر می‌گرفت که از لحاظ آماری معنی‌دار نبود. نتیجه‌گیری: نقاط پرخطر با استفاده از آماره کاوشی فضا – زمان شامل استان‌های شمالی، شمال‌غربی و مرکزی خصوصاً اردبیل، کردستان، مازندران و گیلان می‌باشد. پیشنهاد می‌شود آزمایشات غربالگری در نقاط پرخطر و مطالعات اپیدمیولوژیک بیشتری برای پیاده‌سازی برنامه‌های پیشگیری از آن صورت پذیرد.

Title: بررسی عوامل مؤثر بر دفع پیوند قرنیه در بیماران قوز قرنیه با بکارگیری مدل‌بندی بیزی خطر وایبول دومتغیره بر اساس تابع مفصل کلایتون

http://hsr.mui.ac.ir/index.php/jhsr/article/view/2167

مقدمه: کراتوکنوس یک بیماری دو طرفه قرنیه است، که یکی از راه‌های درمان آن انجام پیوند است. ممکن است پیوند توسط سیستم ایمنی فرد گیرنده، دفع گردد که این مسأله موجب شکست پیوند خواهد شد. هدف این مطالعه تحلیل بیزی عوامل مؤثر بر دفع پیوند قرنیه دو طرفه بر اساس تابع مفصل است. روش‌ها: یک نمونه از زمان بقای پیوند دوطرفه کراتوکنوس بررسی شد. از آن‌جایی که بین زمان‌های دفع پیوند قرنیه همبستگی وجود دارد، برای تحلیل داده‌های زمان دفع پیوند قرنیه دو طرفه از مفصل ارشمیدسی کلایتون استفاده شد، به طوری‌که برآورد پارامترها به روش تحلیل بیزی و بر اساس الگوریتم‌های مونت‌کارلوی زنجیر مارکوفی و به کمک نرم‌افزار WinBUGS انجام گرفت. برای مدل‌بندی تابع خطر، مدل خطر وایبول به‌کار گرفته شد. یافته‌ها: وجود واسکولاریزاسیون قرنیه در زمان دفع پیوند اول و تازگی قرنیه در زمان دفع پیوند دوم تأثیر معنی‌داری بر زمان دفع پیوند داشتند. هم‌چنین سن در زمان انجام پیوند، تأثیر جزیی معنی‌دار در هر دو زمان دفع اول و دوم داشت. میانگین پسین پارامتر همبستگی برای مفصل کلایتون برابر با 98/0 و ضریب همبستگی تاوکندال بین زمان‌های دفع پیوند اول و دوم قرنیه 3/0 به دست آمد. نتیجه‌گیری: نتایج حاکی از همبستگی بین زمان‌های دفع پیوند اول و دوم است. افزایش سن به‌صورت جزیی خطر دفع پیوند را بالا می‌برد. نگهداری قرنیه در محلول و هم‌چنین وجود واسکولاریزاسیون قرنیه باعث افزایش خطر دفع پیوند قرنیه می‌شود..

Title: غذاهای گرم برمبنای عقاید مردم

http://hsr.mui.ac.ir/index.php/jhsr/article/view/1419

مقدمه: غذاهای فراویژه و یا غذاهای عملکردی از دسته غذا- داروها محسوب می‌شوند که دارای خواص درمانی چشمگیری در پیشگیری و درمان انواع بیماری‌های دژنراتیو وسرطان‌ها می‌باشند. ترکیبات بیواکتیو اقلام مختلف غذایی متناسب بامزاج ذاتی بدن، روش زندگی، عملکرد جسمی و روحی و هم‌چنین اصول و مبانی طب سنتی می‌تواند در کنترل اختلالات مزمن و هزینه‌های اقتصادی مثمر ثمر واقع شود. بدین منظور مطالعه حاضر به بررسی باورها و عقاید مردم در خصوص خواص غذا- داروها برمبنای تأثیر سردی، گرمی آن‌ها می‌پردازد. روش‌ها: این مطالعه مقطعی بر700 زن خانه‌دار اصفهانی از اردیبهشت تا بهمن ماه سال 90 اجراشده است. نمونه‌گیری باروش خوشه‌ای سیستماتیک انجام شد. با استفاده از پرسش‌نامه نظرات و باورهای شرکت‌کنندگان درخصوص خواص غذاها از دیدگاه طب سنتی مورد بررسی قرار گرفت و سپس با مستندات معتبر طب سنتی ایران از جمله کتب "قانون در طب"، "الشامل فی الصناعه الطیبه" و "مخزن الادویه" مقایسه گردید. آنالیز داده‌ها با استفاده از جدول اعداد متقاطع و تست آماری مک نمار در نرم‌افزار SPSS نسخه 20 اجرا شد. یافته‌ها: نتایج حاصل از بررسی‌ها نشان داد که30 نوع از اقلام و فرآورده‌های غذایی عمدتا از دسته ادویه و چاشنی‌ها، میوه‌جات، انواع گوشت‌ها و انواع منابع کربوهیدرات‌های ساده و مواد شیرین‌کننده بودند که این اقلام مذکور تحت عنوان غذاهای با طبع گرم از نظر عموم شرکت‌کنندگان مطرح گردیدند. نتیجه حاصل از مقایسه‌ها حاکی از تطابق حدودا 50 درصدی اعتقادات زنان با مستندات مرتبط طب سنتی بود. نتیجه‌گیری: بررسی درصد توافق خواص مواد غذایی با مستندات، بیانگر لزوم تمرکز بر آموزش و فراهم‌سازی زمینه شناخت خواص غذا– داروها در زنان به عنوان مسؤول تغذیه خانواده می‌باشدکه می‌تواند در راستای پیشگیری، کنترل و درمان بیماری‌ها مفید واقع گردد.

Title: میزان خودکارآمدی پژوهشی در بین دانشجویان دانشگاه علوم پزشکی اصفهان براساس مقیاس خودکارآمدی پژوهشی

http://hsr.mui.ac.ir/index.php/jhsr/article/view/1812

مقدمه: يكي از مهم‌ترين عوامل مؤثر بر انجام تحقيق در دانشجويان، باورهاي آن‌ها نسبت به توانايي‌هايشان در اين زمينه است. هم‌چنین آگاهی اساتید و مربیان از خودکارآمدی پژوهشی دانشجویان، می‌تواند نقش به‌سزایی در تربیت دانشجویان پژوهشگر داشته باشد. هدف این پژوهش، تعیین میزان خودکارآمدی پژوهشی در بین دانشجویان دانشگاه علوم پزشکی اصفهان براساس مقیاس خودکارآمدی پژوهشی می‌باشد. روش‌ها: روش پژوهش، پیمایشی تحلیلی و نوع مطالعه مقطعی است که در سال 1391انجام گرفت. جامعه آماری، همه دانشجویان دانشگاه علوم پزشکی اصفهان می‌باشد که بر اساس جدول کرجسی و مورگان حجم نمونه 344 دانشجو به دست آمد. روش نمونه‌گیری تصادفی طبقه و سهم هر دانشکده یکسان (44 نفر) بود. ابزار گردآوری اطلاعات پرسش‌نامه استاندارد (شامل 7 بعد) با طیف لیکرت (5 گزینه‌ای) است. روش گردآوری اطلاعات مراجعه حضوری به دانشکده‌های مذکور بوده است. نوع آمار توصیفی و استنباطی (تحلیل واریانس یک طرفه و t مستقل) و نرم‌افزار مورد استفاده جهت تجزیه و تحلیل داده ها SPSS نسخه 21 می‌باشد. یافته‌ها: یافته‌ها نشان داد، بعد اخلاق پژوهش بیشترین میانگین و بعد پژوهش کیفی کمترین میانگین را در بین ابعاد خودکارآمدی پژوهشی به خود اختصاص داده‌اند. هم‌چنین مقایسه نمره کلی خودکارآمدی پژوهشی با ویژگی‌های جمعیت‌شناختی حاکی از این بود که تفاوت معنی‌داری بین نمره کلی خودکارآمدی پژوهشی با جنس، دانشکده و مقطع تحصیلی وجود ندارد (05/0<p) نتیجه‌گیری: از آن‌جا که نمره اکثر ابعاد خودکارآمدی پژوهش پایین‌تر از حد متوسط بوده است، لذا برگزاری کار‌گاه‌های مناسب به صورت دوره‌ای و متناوب، گنجاندن واحدهای درسی در سرفصل دروس، موظف کردن دانشجویان به انجام طرح‌ها و مقالات پژوهشی در مقاطع تحصیلی پایین و نهادینه کردن فرهنگ تحقیق برای دانشجویان توسط اساتید مجرب، از جمله راهکارهای عملی است که می‌تواند انگیزه لازم را برای انجام پژوهش کارآمد توسط دانشجویان به همراه داشته باشد.

Title: بررسی میزان آگاهی، نگرش و رفتار دانشجویان دانشگاه علوم پزشکی شیراز درباره ‏بازیافت مواد زائد جامد در سال 1391‏

http://hsr.mui.ac.ir/index.php/jhsr/article/view/1573

مقدمه: مواد زائد جامد طی فعالیت‌های مختلف انسان تولید می‌شود که مدیریت مطلوب آن نیازمند داشتن آگاهی و نگرش همگانی در این زمینه است. هدف این مطالعه تعیین ‏میزان آگاهی، نگرش و رفتار دانشجویان دانشگاه علوم پزشکی شیراز در رابطه با بازیافت مواد ‏زائد جامد است.‏ روش‌ها: این مطالعه مقطعی- تحلیلی بر روی 383 دانشجوی دانشگاه علوم پزشکی ‏شیراز به روش نمونه‌گیری طبقه‌ای انجام شده ‏است. ابزار جمع‌آوری اطلاعات پرسش‌نامه‌ای که حاوی 3 سؤال مربوط به بخش دموگرافیک، 12 سؤال ‏مربوط به بخش آگاهی، 7 سؤال مربوط به سؤالات نگرشی و 5 سؤال مربوط به حیطه رفتار بودند. ‏داده‌ها پس از جمع‌آوری، توسط نرم افزار ‏SPSS 16‎‏ تجزیه و تحلیل شدند.‏ یافته‌ها: میانگین نمره آگاهی دانشجویان (میانگین 0 تا 20 امتیاز کل) برابر با 97/2±59/12 و به ‏ترتیب در میان دختران 80/2± 18/12 و پسران 15/3±47/13گزارش شد (‏‎001/0P<‎‏). میانگین ‏نمره نگرش دانشجویان (میانگین 7 تا 35 امتیاز کل)20/3±56/13 بود که برای دانشجویان دختر برابر با ‏‏39/3± 74/13 و برای پسران 73/2 ±8/13بود (‏2/ 0P=‎‏). میانگین نمره رفتار دانشجویان (میانگین 4 تا 8 ‏امتیاز کل) برابر با 36/1±13/6 و به ترتیب در میان دختران 37/1 ±11/6 و در پسران 34/1± 16/6 ‏بود (‏74/0P=‎‏). نتیجه‌گیری: آگاهی و رفتار دانشجویان در رابطه با موضوع مورد بررسی، در سطح متوسطی بود. بنابراین با توجه به اهمیت آموزش مؤثر مسایل بهداشتی و زیست محیطی، نیاز به برنامه‌ریزی مناسبی برای ارتقا آگاهی و رفتار دانشجویان در مورد مدیریت مواد زائد به ویژه کاهش تولید و بازیافت پسماندها می‌باشد.

Title: عوامل مؤثر بر سکوت سازماني کارکنان (مورد مطالعه: بیمارستان حضرت زهرا (س) اصفهان)

http://hsr.mui.ac.ir/index.php/jhsr/article/view/888

مقدمه: امروزه با توجه به دگرگوني‎هاي صورت گرفته در سازمان‎ها و محيط آن‌ها، اهميت منابع انساني افزايش يافته است. يکي از مسایل مطرح در اين زمينه پديده سکوت سازماني مي‌باشد. اين پژوهش با هدف بررسي نحوه تأثير خودپنداره، هويت سازماني و ارزش‎هاي سازماني بر سکوت سازماني و هم‌چنين بررسي نقش تعديل‌کنندگي متغير خودپنداره و ارزش‎هاي سازماني بر رابطه بين هويت سازماني و سکوت سازماني در بين کارکنان بیمارستان حضرت زهرا (س) اصفهان انجام شده است. روش‌ها: اين پژوهش از نوع توصيفي است و شامل 5 فرضيه مي‌باشد که براي بررسي اين فرضيات از پرسش‌نامه محقق ساخته که شامل 49 سؤال بود، استفاده گرديد. ضريب پايايي کلي پرسش‌نامه 912/0به دست آمد که از پايايي لازم برخوردار بود. حجم جامعه آماري مورد نظر 160 نفر مي‌باشد. لذا بر اساس روش نمونه‌گيري تصادفي از بين آن‎ها نمونه‎ي آماري انتخاب گرديد و در نهايت 70 پرسش‌نامه جمع‌آوري گرديد. هم‌چنین از نرم‌افزار Spss18 برای تجزیه و تحلیل داده‌ها استفاده شد. یافته‌ها: نتايج حاصل از پژوهش حاکي از آن است که سکوت سازماني تحت تأثير هويت سازماني، خودپنداره و ارزش‎هاي سازماني قرار دارد و هر کدام از آن‌ها داراي همبستگي منفي با سکوت سازماني مي‎باشند. هم‌چنين نتايج نشان داد که خودپنداره رابطه بين هويت سازماني و سکوت سازماني را تعديل مي‎کند ولي ارزش‎هاي سازماني نقش تعديل‌کنندگي در اين رابطه ندارند. نتیجه‌گیری: با تقويت هويت سازماني، خودپنداره مثبت کارکنان و ارزش‌های سازمانی مي‌توان سکوت سازماني را در ميان افراد کاهش داد.
